# Thrombotic Thrombocytopenic Purpura in the Setting of Cirrhosis and Baseline Thrombocytopenia

**DOI:** 10.7759/cureus.59839

**Published:** 2024-05-07

**Authors:** Priyanka Venkatesh, Joseph Bennett, Konstantine Halkidis

**Affiliations:** 1 Department of Internal Medicine, University of Kansas Medical Center, Kansas City, USA; 2 Department of Hematologic Malignancies and Cellular Therapeutics, University of Kansas Medical Center, Kansas City, USA

**Keywords:** ttp, thrombocytopenia, splenomegaly, cirrhosis, adamts13

## Abstract

The management of immune thrombotic thrombocytopenic purpura (iTTP) has evolved significantly over the past several years. However, despite recent advances, there are limited tools available for patients with comorbidities that preclude either the utilization of available treatment modalities or evidence-based laboratory target levels. Literature to guide the management of such patients is sparse at best, and many complications associated with pre-existing comorbidities in the context of iTTP have not been reported. Here we describe the case of a patient with severe thrombocytopenia at baseline due to liver cirrhosis who developed iTTP. The challenges of the case in terms of pursuing disease-directed treatment, defining laboratory parameters to guide treatment, and mitigating the risks of bleeding and disease exacerbation are discussed. We offer our perspective in treating iTTP in the setting of severe baseline thrombocytopenia and high bleeding risk.

## Introduction

Thrombotic thrombocytopenic purpura (TTP) is characterized by microangiopathic hemolytic anemia, severe thrombocytopenia, and organ ischemia linked to disseminated microvascular platelet-rich thrombi [1]. Immune TTP (iTTP) is a type of thrombotic microangiopathy caused by antibody-mediated severe deficiency of a disintegrin and metalloproteinase with a thrombospondin type 1 motif, member 13 (ADAMTS13), which plays the central role in von Willebrand factor (VWF) multimer regulation [2]. Many treatment options have been described for iTTP including plasma exchange, steroids, rituximab, salvage splenectomy, and caplacizumab; usually, a normal platelet count is the target of iTTP-directed therapy [3]. However, thrombocytopenia due to other causes can complicate the diagnosis and treatment of iTTP. Additionally, though iTTP is a thrombotic disease, available treatments can and do increase the risk of hemorrhagic complications, particularly for patients who already have a high bleeding risk due to baseline thrombocytopenia [4]. Our case presents the unique conundrums associated with the management of iTTP in a patient with pre-existing liver cirrhosis and severe thrombocytopenia at baseline.

## Case presentation

A 62-year-old male with decompensated alcoholic Child-Pugh class B cirrhosis complicated by refractory ascites, grade 2 esophageal varices with variceal bleeding treated with banding, and a recent episode of spontaneous bacterial peritonitis (SBP) presented to a community hospital with complaints of headaches, light-headedness, and a fall at home. Past medical history was also pertinent for a recent non-ST-elevation myocardial infarction (NSTEMI) with 70% left anterior descending (LAD) artery lesion on medical therapy. Initial workup revealed a platelet count of 8000/µL, compared to a baseline platelet count of around 30,000-40,000/µL in the setting of cirrhosis. CT head was negative for acute intracranial hemorrhage. The patient was transfused platelets and underwent paracentesis; 4.5 liters of bloody ascitic fluid was removed which was negative for SBP and cytology was negative for malignancy. CT of the abdomen showed scattered splenic hypodensities concerning for infarction which were new compared to imaging completed 14 months prior. The patient was then transferred to our hospital for further management. Hepatology and hematology were consulted. MRI brain showed small acute infarcts throughout the right and left cerebral hemispheres and the right cerebellum. He was started on high-dose dexamethasone (40 mg) for presumed ITP. The patient was not a candidate for thrombopoietin because of prior known superior mesenteric vein (SMV) occlusion. Additional laboratory studies showed elevated international normalized ratio (INR) (1.6), fibrinogen 174 mg/dL (reference range 200-400 mg/dL), lactate dehydrogenase (LDH) 377 U/L (reference range 100-210 U/L), immature platelet fraction 9.4% (reference range 1-7%) and negative Coombs test. Peripheral smear showed moderate macrocytic anemia and thrombocytopenia, with increased bite cells, spur cells, and 5-7 schistocytes per high power field. At this point, steroids were stopped. The calculated PLASMIC score was 5 [5]. ADAMTS13 testing was then performed in-house; activity resulted within 24 hours and was <1 IU/dL (reference range 40-133 IU/dL). iTTP-directed therapy was initiated with therapeutic plasma exchange (TPE) and methylprednisolone at a dose of 125 mg IV every six hours for 48 hours. Within 48 hours ADAMTS13 IgG antibody level was found elevated at 87 units/mL (reference range <12 units/mL). The use of caplacizumab was considered but the decision was made to avoid this agent due to bleeding risk with cirrhosis and esophageal varices, as well as the presence of bloody ascites and the patient’s history of variceal bleeding. Platelet count improved from 9000/µL to 34,000/µL and repeat ADAMTS13 activity was 25 IU/dL after five sessions. Anti-CD20 therapy was pursued, and the patient received the first of four planned doses of rituximab IV 375 mg/m^2^ after the fourth TPE treatment.

**Figure 1:**
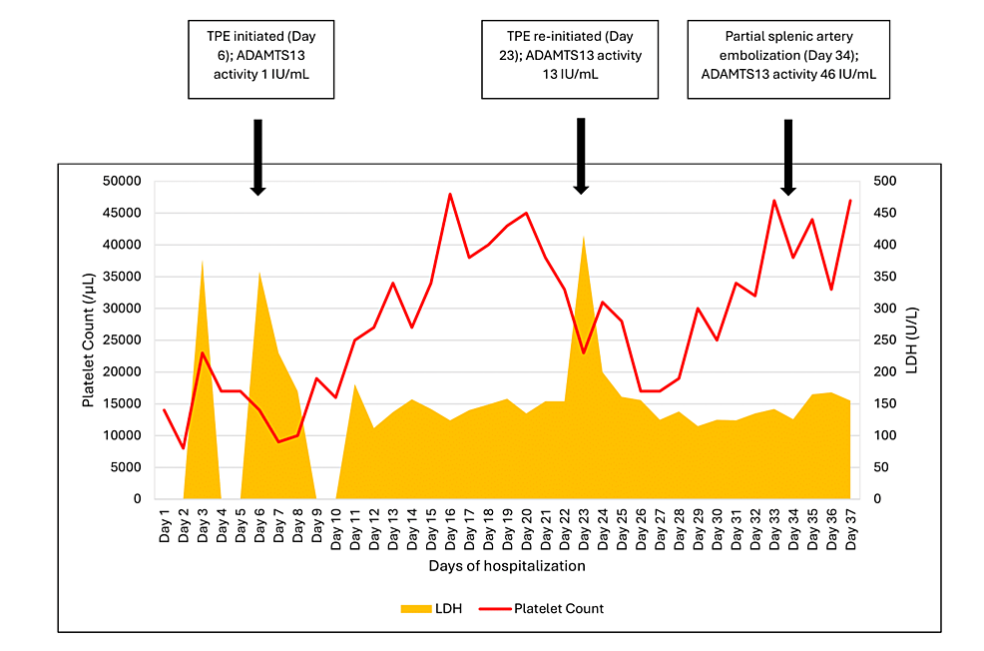
Timeline of events. Platelet count and lactate dehydrogenase level on each day of inpatient hospitalization are graphically depicted. Selected ADAMTS13 levels reported along with significant events as arrows on the timeline. LDH: lactate dehydrogenase; TPE: therapeutic plasma exchange

Given the complexity of the scenario, the case was discussed at our internal hematology conference and consensus target laboratory parameters were defined. TPE continued until platelet count was >40,000/µL and LDH remained within the normal range for two consecutive days. Platelet count began to drop once again five days after stopping TPE; LDH rose to 415 U/L, haptoglobin was <30 mg/dL, and repeat ADAMTS13 activity dropped to 13 IU/dL. TPE was restarted. To address the patient’s baseline thrombocytopenia and to better determine the effect of TTP management, interventional radiology (IR) was engaged to evaluate the patient’s candidacy for partial splenic artery embolization. Before embolization, platelet transfusion was requested given the procedural risks. Embolization was thus delayed until the second round of TPE was complete and repeat ADAMTS13 levels were obtained. The repeat ADAMTS13 level was 46 IU/dL three days after completing the second round of TPE. Embolization was performed without complication. The patient received one unit of platelets immediately before the procedure. Platelet count improved to 44,000/µL prior to discharge (Figure 1). Four total doses of rituximab were given during the hospitalization. Steroids were continued during hospitalization with a planned slow prednisone taper over six to eight weeks. The patient was discharged with close outpatient follow-up with a local hematologist with bi-weekly labs. The central line was left in situ on discharge in case of iTTP exacerbation.

Outcomes and follow-ups

The patient continued the prednisone taper. Platelet count was 40,000/µL two months after discharge and improved to 75,000/µL at three-month follow-up. The patient continues to receive weekly paracentesis and is currently being evaluated for a liver transplant at our center.

## Discussion

Treatment of iTTP is based on a combination of clinical and laboratory features and risk-benefit analysis for each patient. TPE remains the mainstay of treatment, used in patients with a presumptive diagnosis of TTP based on an intermediate to high PLASMIC score (5-7 points) or if high clinical suspicion of iTTP. An important tenet of management is that the decision for plasma exchange should not be delayed while awaiting laboratory confirmation with ADAMTS13 level if rapid testing is not available, as this can delay appropriate therapy. Once the decision is made to start treatment, the outcome can be determined based on guidelines set by the International Society of Thrombosis and Hemostasis (ISTH) and the International Working Group Consensus Report [3,6]. Response to treatment is defined as a sustained platelet count ≥150,000/µL and LDH <1.5 times the upper limit of normal (ULN) and no clinical evidence of new or progressive ischemic organ injury. The generally accepted definition of exacerbation or relapse of iTTP is a decrease in platelet count to <150,000 /µL after completion of TPE and/or anti-VWF therapy, which must be confirmed by documentation of ADAMTS13 deficiency.

Cirrhosis and concomitant severe thrombocytopenia at baseline posed unique challenges to iTTP-directed treatment in our patient in terms of defining laboratory-based treatment targets. Another aspect to consider was the correlation between ADAMTS13 activity and liver dysfunction. ADAMTS13 is encoded by a gene located on chromosome 9q34 and is primarily expressed in hepatic stellate cells [7]. Patients with advanced cirrhosis have decreased ADAMTS13 activity which might reflect a predisposition for platelet thrombi formation [8]. A subclinical TTP-like state without disseminated intravascular coagulation (DIC) has also been described in these patients. However, very low ADAMTS13 activity, as seen in TTP, is exceedingly rare even among patients with essentially absent hepatic function [9-12]. As such, patients with findings of severe ADAMTS13 deficiency in the setting of microangiopathic hemolytic anemia should be treated for iTTP, even in the setting of Child-Pugh class C cirrhosis, considering the high mortality risk of leaving iTTP untreated.

The ISTH also recommends the use of caplacizumab, a nanobody that targets the VWF A1 domain and thus prevents platelet binding to VWF, in acute iTTP [4,13]. Indeed, evolving data has shown that the use of caplacizumab confers a decreased risk of morbidity and mortality associated with iTTP [14]. However, caplacizumab does increase bleeding risk. Patients with advanced cirrhosis have an increased risk of hemorrhagic complications [15]. The presence of varices further compounds this risk.

While splenic embolization has been extensively studied in patients with cirrhosis to improve hypersplenism, its role in TTP is less clear [16]. Splenectomy or partial splenectomy is associated with a risk of operative morbidity and mortality, intraperitoneal hemorrhage, infection, venous thromboembolism, ischemic heart disease, and pulmonary hypertension [17,18]. As there is an increased risk for infections, pre-operative vaccinations are now recommended for all patients. Further complicating the scenario, rituximab reduces the success rates of vaccinations [19]. With both splenic artery embolization and splenectomy, clinicians must carefully weigh the risks of procedures, long-term infection risks, and potential benefits for patient outcomes.

Current evidence does not support routine platelet transfusions in patients with TTP. A recent study on 10635 admissions for TTP between 2007 and 2011 showed that platelet transfusions were associated with an increased risk of arterial thrombosis (OR=5.8, 95% CI=1.3-26.6) and acute myocardial infarction (OR=2.0, 95%CI=1.2-3.3) [20]. To mitigate the risks of platelet transfusion prior to splenic artery embolization, we aimed to normalize ADAMTS13 activity as much as possible prior to proceeding. This approach has not been previously described and might help guide decision-making in patients with iTTP and baseline thrombocytopenia due, at least in part, to splenomegaly.

## Conclusions

This case illustrates our experience with the treatment of iTTP in the setting of severe baseline thrombocytopenia due to liver cirrhosis. We aim to highlight the challenges of treatment for such patients, with sparse available literature to guide clinical decision-making. The use of adjunctive therapies such as splenic artery embolization in our patient also played an important role in improving platelet counts and should be considered in every patient as clinically appropriate. Further research is necessary to optimize the care of iTTP patients with cirrhosis, baseline thrombocytopenia, and/or high bleeding risk.

## References

[1] George JN, Al-Nouri ZL (2012). Diagnostic and therapeutic challenges in the thrombotic thrombocytopenic purpura and hemolytic uremic syndromes. Hematology Am Soc Hematol Educ Program.

[2] Zheng X, Majerus EM, Sadler JE (2002). ADAMTS13 and TTP. Curr Opin Hematol.

[3] Zheng XL, Vesely SK, Cataland SR (2020). ISTH guidelines for treatment of thrombotic thrombocytopenic purpura. J Thromb Haemost.

[4] Scully M, Cataland SR, Peyvandi F (2019). Caplacizumab treatment for acquired thrombotic thrombocytopenic purpura. N Engl J Med.

[5] Kim CH, Simmons SC, Williams LA II, Staley EM, Zheng XL, Pham HP (2017). ADAMTS13 test and/or PLASMIC clinical score in management of acquired thrombotic thrombocytopenic purpura: a cost-effective analysis. Transfusion.

[6] Cuker A, Cataland SR, Coppo P (2021). Redefining outcomes in immune TTP: an international working group consensus report. Blood.

[7] Uemura M, Tatsumi K, Matsumoto M (2005). Localization of ADAMTS13 to the stellate cells of human liver. Blood.

[8] Uemura M, Fujimura Y, Matsumoto M (2008). Comprehensive analysis of ADAMTS13 in patients with liver cirrhosis. Thromb Haemost.

[9] Takaya H, Namisaki T, Asada S (2022). ADAMTS13, vWF, and endotoxin are interrelated and associated with the severity of liver cirrhosis via hypercoagulability. J Clin Med.

[10] Takaya H, Uemura M, Fujimura Y (2012). ADAMTS13 activity may predict the cumulative survival of patients with liver cirrhosis in comparison with the Child-Turcotte-Pugh score and the Model for End-Stage Liver Disease score. Hepatol Res.

[11] Reuken PA, Kussmann A, Kiehntopf M, Budde U, Stallmach A, Claus RA, Bruns T (2015). Imbalance of von Willebrand factor and its cleaving protease ADAMTS13 during systemic inflammation superimposed on advanced cirrhosis. Liver Int.

[12] Simbrunner B, Villesen IF, Scheiner B (2023). Von Willebrand factor processing in patients with advanced chronic liver disease and its relation to portal hypertension and clinical outcome. Hepatol Int.

[13] Peyvandi F, Scully M, Kremer Hovinga JA (2017). Caplacizumab reduces the frequency of major thromboembolic events, exacerbations and death in patients with acquired thrombotic thrombocytopenic purpura. J Thromb Haemost.

[14] Peyvandi F, Cataland S, Scully M (2021). Caplacizumab prevents refractoriness and mortality in acquired thrombotic thrombocytopenic purpura: integrated analysis. Blood Adv.

[15] Kujovich JL (2015). Coagulopathy in liver disease: a balancing act. Hematology Am Soc Hematol Educ Program.

[16] Hadduck TA, McWilliams JP (2014). Partial splenic artery embolization in cirrhotic patients. World J Radiol.

[17] Crary SE, Buchanan GR (2009). Vascular complications after splenectomy for hematologic disorders. Blood.

[18] Kristinsson SY, Gridley G, Hoover RN, Check D, Landgren O (2014). Long-term risks after splenectomy among 8,149 cancer-free American veterans: a cohort study with up to 27 years follow-up. Haematologica.

[19] Kimby E (2005). Tolerability and safety of rituximab (MabThera). Cancer Treat Rev.

[20] Goel R, Ness PM, Takemoto CM, Krishnamurti L, King KE, Tobian AA (2015). Platelet transfusions in platelet consumptive disorders are associated with arterial thrombosis and in-hospital mortality. Blood.

